# Evaluation of Effective Condyle Positioning Assisted by 3D Surgical Guide in Mandibular Reconstruction Using Osteocutaneous Free Flap

**DOI:** 10.3390/ma13102333

**Published:** 2020-05-19

**Authors:** Seong Ryoung Kim, Sam Jang, Kang-Min Ahn, Jee-Ho Lee

**Affiliations:** 1 Department of Oral and Maxillofacial Surgery, College of Medicine, University of Ulsan, ASAN MEDICAL CENTER, Seoul 05505, Korea; alwaysyouth37@gmail.com (S.R.K.); ahnkangmin@hanmail.net (K.-M.A.); 2 Coreline Soft, Seoul 03991, Korea; semyeong.jang@corelinesoft.com

**Keywords:** three dimensional printing, microvascular flap, mandible reconstruction

## Abstract

In the present study, the reproducibility and postoperative stability of a 3D printed surgical guide were evaluated in mandibular reconstruction with an osteocutaneous free flap (OCFF), including a fibular free flap (FFF) and deep circumflex iliac artery free flap (DCIA). Fifteen patients were enrolled, and a 3D surgical guide was fabricated by simulation surgery using preoperative (T0) Computed tomography (CT) images. Mandibular reconstruction was performed with OCFF using the 3D surgical guide. Postoperative CTs were taken immediately, 1 week (T1), and 6 months (T2) after surgery, to evaluate the reproducibility of the 3D surgical guide and condyle stability. Error of the 3D surgical guide ranged from 0.85 to 2.56 mm. There were no differences in reproducibility according to flap type. Condylar error and error at mandible midpoint were significantly different in FFF. However, there was no difference in DCIA error between the condyle and mandible midpoint. Regarding condyle stability 6 months after surgery, condyles moved more than 2 mm (up to 2.85 mm) in FFF, whereas there were no significant movement in DCIA. Careful intraoperative flap fixation and closed postoperative observation should be considered for stable clinical outcome, especially in the case of FFF.

## 1. Introduction

The mandible is an important structure in the maxillofacial area that functions in mastication, pronunciation, and organization of the lower facial contour [1,2]. Mandible resection is usually indicated in oral cancer, trauma, and severe osteomyelitis, which can eventually result in large composite tissue defect of tooth, oral mucosa, and mandibular bone [3,4]. Subsequent reconstruction of mandibular defects should consider the restoration of function and esthetics of maxillofacial structure, to improve patient quality of life [1]. Reconstruction of the mandible with vascularized osteocutaneous free flap (OCFF) has become the gold standard method for patients undergoing mandibular resection because it allows composite tissue reconstruction of wide mandibular defect. This technique maintains viability under postoperative radiation therapy and provides enough bone for future dental implant rehabilitation. There are several options for mandibular reconstruction with OCFF, including fibula free flap (FFF), deep circumflex iliac artery free flap (DCIA), and scapula free flap [5,6]. FFF and DCIA are popular options for mandible reconstruction [2,7]. FFF involves a long cortical bone shaft, which is suitable for wide resection of mandible. Moreover, it can be harvested with a wide skin paddle that can be used for oral mucosa or facial skin defect [8,9,10]. However, it has a smaller height than the mandibular body, and is relatively unfavorable for dental implant rehabilitation. DCIA has some advantages compared with FFF, which can have a similar height as the mandibular body, and provide sufficient bone for dental implant. In addition, its natural curve can reproduce the original contour of the mandible [2,7,11]. Lonie et al. recommended that DCIA is an option for mandible body or angle, whereas FFF should be considered for total or subtotal mandibulectomy [2].

With the advent of virtual surgery based on computer simulation software and 3D printing technology, 3D surgical guides for OCFF can be designed before performing surgery. Various surgical simulation processes and 3D printed surgical guides have been introduced for clinical applications in mandibular reconstruction [12,13,14]. Reconstructive surgery has been easily guided by the use of intraoperative 3D printed surgical guides [15,16,17,18]. De Maesschalck et al. reported that 3D print assisted reconstruction and conventional manual technique are comparable for mandibular reconstruction with FFF and provide satisfactory mandibular contour [17]. However, intraoperative condyle repositioning is a critical procedure for mandibular reconstruction with OCFF and repairing original mandible contour. Additionally, the mandibular condyle should maintain its own original position. Condyle displacement may induce temporomandibular joint problems, such as limited mouth opening, pain, clicking, malocclusion, or bone segment breakage, which threaten good clinical outcomes [19]. However, to our knowledge, there are few studies on microvascular reconstruction of the mandible using 3D surgical guides that focused on condylar stability after surgery. Therefore, the present study evaluated the reproducibility of original condyle position and postoperative stability in mandibular reconstruction with OCFF, using a 3D surgical guide.

## 2. Materials and Methods

### 2.1. Patients

Inclusion criteria in this retrospective study were mandibular reconstruction using vascularized OCFF 1) with 3D surgical guide, 2) defect within subcondyle and midline of mandible, and 3) without clinical complication for one year follow up. Two cases were excluded, as one was immediate failure of flap, due to venous thrombosis, and the other was early recurrence of malignancy. Fifteen patients met inclusion criteria among 27 patients who had mandibular reconstruction, due to head and neck cancer, osteomyelitis, or trauma from 2016 to 2018 in the department of oral and maxillofacial surgery of the Asan Medical Center (Table 1). The study protocol was reviewed and approved by the institutional review board of the Asan Medical Center, Seoul, Korea (IRB approval No. 2019-1448).

### 2.2. Fabrication of 3D Surgical Guide

**Figure 1:**
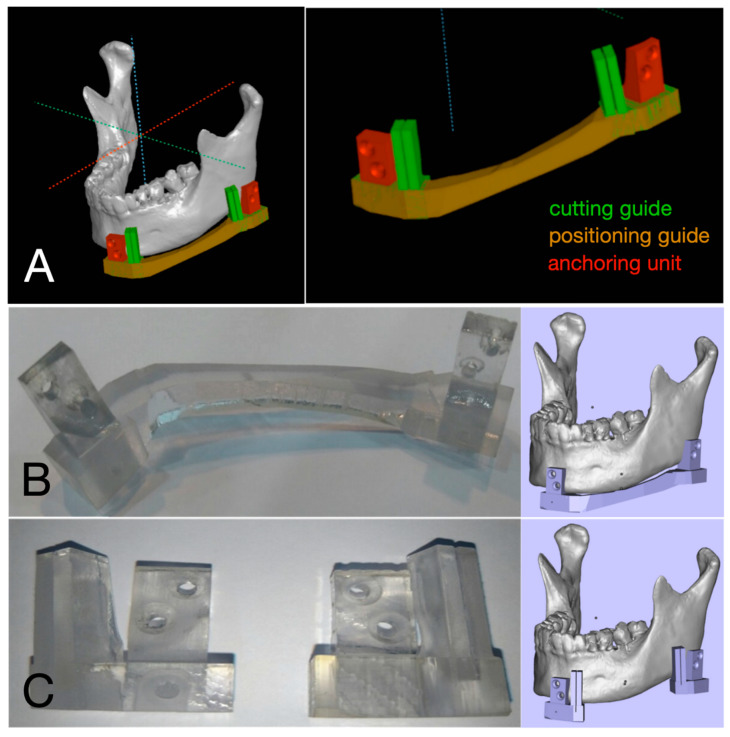
Virtual surgical planning and fabrication of 3D surgical guide. (**A**) Positioning and cutting guide were designed on virtual 3D model. (**B**) Positioning guide combined with anchoring units. (**C**) Cutting guide combined with anchoring units.

Three dimensional Computed tomography (CT) images were taken before surgery. A 3D surgical guide composed of positioning guide, cutting guide, and anchoring unit was designed after original condyle position and occlusion were confirmed on the 3D modeling software platform using preoperative CT images (T0) (Aview(R) Modeler; Coreline Soft, Seoul, Korea or OnDemand3D^®^; Cybermed, Seoul, Korea). The 3D surgical guide was fabricated using 3D printing technology, Polyjet 3D printer (Stratasys Ltd., Eden Prairie, MN, USA). The material used for printing was MED610 (Stratasys Ltd., Eden Prairie, MN, USA), designed for surgical guides and approved for permanent skin and limited mucosal contact (Figure 1 and Supplementary Materials).

### 2.3. Surgical Technique

**Figure 2:**
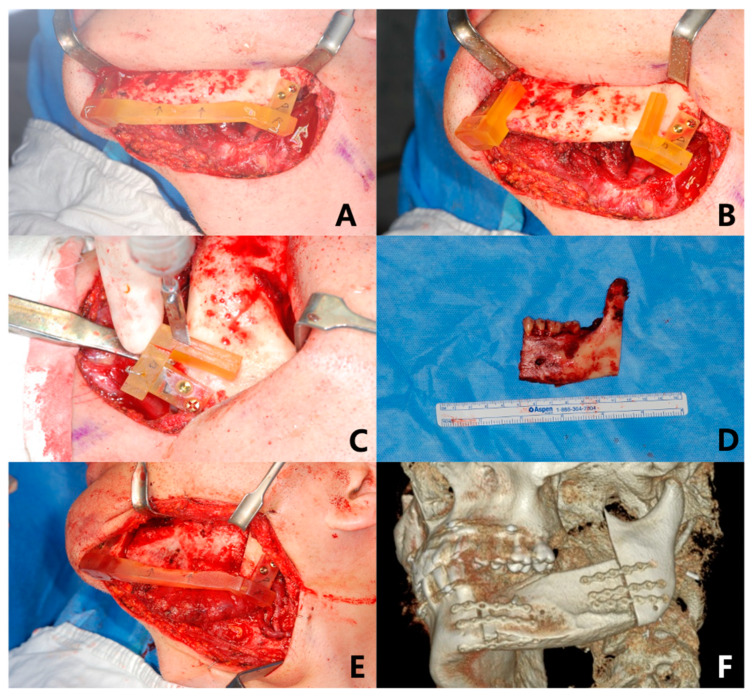
Mandibular reconstruction assisted by 3D surgical guide for case of deep circumflex iliac artery free flap (DCIA). (**A**) Mandibular positioning guide was temporarily fixed before mandibulectomy. (**B**) Cutting guide was engaged to cut mandible as planned on 3D virtual surgery after removal of positioning guide. (**C**) Sawing was performed according to cutting guide. (**D**) Body of mandible with tumor was removed. (**E**) Positioning guide was repositioned, and DCIA was set and fixed while original occlusion and condyle position were maintained. (**F**) Flap position was assessed by postoperative CT.

All cases of reconstruction included in this study were performed by single surgeon (J.-H.L.). The anchoring unit was intraoperatively fixed with temporary combination of the positioning guide on the mandible, to reproduce the original occlusion and condylar position at flap positioning. As the cutting guide was secured in the anchoring unit after removing the positioning guide, osteotomies were performed according to the cutting guide slot to reproduce the virtually planned mandibular resection line. An OCFF (FFF or DCIA) was carefully harvested and tailored, as planned, on a 3D virtual simulation. The flap was positioned into the mandible defect while being repositioned according to the positioning guide, to regain original occlusion and condylar positions. Microvascular anastomoses performed and semi rigid fixation of OCFF were completed using a mini plate system (Figure 2). One week after surgery, light guiding elastic was applied for 6 weeks to maintain original occlusion.

### 2.4. 3D Analysis for Reproducibility of 3D Surgical Guide and Stability of Condylar Position

**Figure 3:**
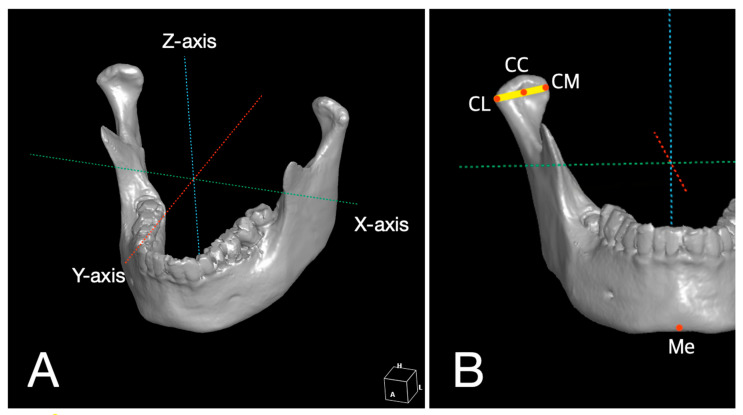
Schematic models of three-dimensional (3D) coordinated systems. (**A**) Positional changes of landmarks were presented as changes (mm) in coordinates (*x*: mediolateral direction, *y*: anteroposterior direction, *z*: superoinferior direction) and distance (mm) (*D* = $\sqrt{x^{2}+y^{2}+z^{2}}$). (**B**) Anatomic landmarks were defined on 3D model reconstructed using CT images in 3D coordinate system (CL = lateral pole of condylar head, CM = medial pole of condylar head, CC = center of mandibular condyle, Me = lowermost point on lateral jaw projection).

Postoperative CTs were taken at 1 week (T1) and 6 months (T2) after surgery, respectively. The positions of each mandible landmark from CT images were recorded digitally as 3D coordinates (*x*, *y*, *z*) using simulation software (OnDemand3D^®^; Cybermed, Seoul, Korea). Positional changes according to time are presented as changes (mm) in coordinates (*x*: mediolateral direction, *y*: anteroposterior direction, *z*: superoinferior direction) and distance (mm, *D* = $\sqrt{x^{2}+y^{2}+z^{2}}$) (Figure 3A). Anatomic landmarks were selected and defined to estimate the stability of flaps as follows. CL (lateral pole of condylar head) was the most prominent point of the lateral surface of the mandibular condyle. CM (medial pole of condylar head) was the most prominent point of the medial aspect of the mandibular condyle. CC (center of mandibular condyle) was the midpoint between CL and CM. ME (lowermost midpoint on lateral jaw projection) was the lowest point of the chin (Figure 3B).

Spatial changes between immediate postoperative and preoperative (T1-T0) scans were calculated to assess the reproducibility of the surgical guide. The difference between T2-T0 (change 6 months after surgery) and T1-T0 (change immediately after surgery) was calculated to assess the stability of condylar position.

### 2.5. Statistical Analysis

All statistical analyses were performed using SPSS statistical software (Version 21, IBM Corp, Armonk, NY, USA). Results were presented as mean (standard deviation) in millimeters. The positional changes in landmarks between preoperative, immediate postoperative, and 6-month follow-up stages were assessed using the Wilcoxon signed rank test for continuous variables. A p-value < 0.05 was considered significant.

## 3. Results

The mean patient age was 50.1 ± 19.9 years in the DCIA group and 69.0 ± 15.6 years in the FFF group (Table 1). Male patients were more likely to undergo surgery than female patients in both groups (six vs. four in the DCIA group and three vs. two in the FFF group). However, there were no significant differences in gender and mean age between DCIA or FFF groups (*p* = 0.71 and 0.25, respectively). Designating landmarks was performed by one researcher (S.R.K.). Patients’ information was not presented and time of CT taken (T0, T1, T2) was not informed either. Intra-rater and inter-rater reliability for designating landmarks were measured using the intraclass correlation coefficient, which showed that all values were over 0.979 (Table S1), indicating a high reliability of designating anatomic landmarks.

### 3.1. Reproducibility of 3D Surgical Guide

**Figure 4:**
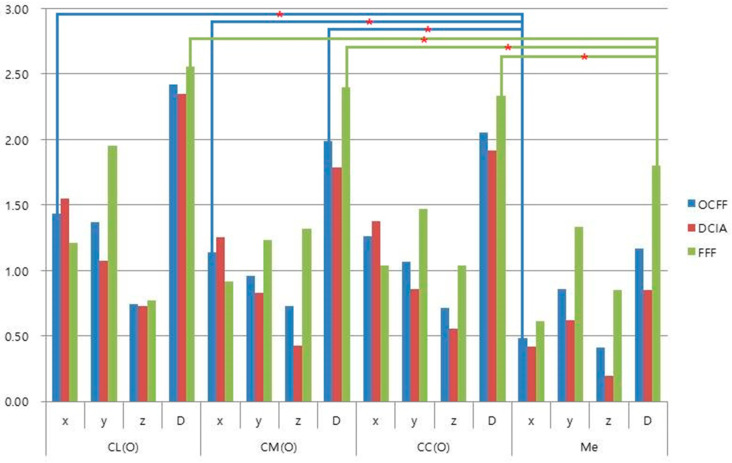
Changes (mm) between preoperative (T0) and immediate postoperative stages (T1). (* *p* < 0.05).

The amount of changes (*D*) between preoperative position (T0) and position immediately after surgery (T1) in the operated side of the mandible ranged from 0.85 mm for the menton with DCIA to 2.56 mm in the lateral pole of the condyle with FFF (Table 2). Although mean changes in FFF were more than those of OCFF and DCIA, there were no significant differences in directions (*x*, *y*, *z*) and the distances (*D*) between T1 and T0, according to the type of flaps in all landmarks (*p* > 0.05). However, condyle landmarks of CL and CM had significant differences with the menton (Me) in the mediolateral direction (*x*) for OCFF and condyle landmarks of CL, CM, and CC, with Me in distance (*D*) for FFF (Figure 4). In the intact side of the mandible, changes between preoperative (T0) and immediate position after surgery (T1) ranged from 0.79 mm in condyle center with DCIA, to 1.12 mm in lateral pole of condyle with FFF (Table S2), and there were no significant differences in landmarks and flap type (*p* > 0.05).

### 3.2. Stability of Condylar Position

With OCFF, the lateral pole of the condyle showed significant movement (1.44 mm) in the mediolateral direction, and the center of the condyle showed significant movement (1.82 mm) in distance (*D*) 6 months after surgery. There was no significant movement in all landmarks of DCIA. FFF has significant movement in all landmarks except the lateral pole of the condyle. This included the mediolateral direction (2.2 mm) in condylar medial pole, mediolateral direction (2.18 mm) and distance (2.85 mm) in center of condyle, and superoinferior direction (1.56 mm) in the menton (Table 3).

## 4. Discussion

Osteocutaneous free flaps have been widely used for mandible reconstruction, and FFF and DCIA are usually chosen as appropriate options [2,12,13]. Elaborate surgical planning for accurate flap positioning should be considered to ensure a successful clinical outcome. However, it is difficult to accurately reproduce the contour and position of the original mandible, because of its three-dimensional structure, movable temporomandibular joint, and complicated occlusion [20,21]. With the recent development of 3D simulation and printing technology, virtual surgical planning for mandibular reconstruction using OCFF has become a safe and reliable technique to increase the accuracy of mandible positioning [20,22,23,24]. A previous systematic review by Rodby et al. reported that the accuracy of reconstruction improved in 93% of cases when comparing virtual and actual osteotomy positions and contours [4]. A virtual surgical plan can optimize surgical results and minimize surgical time. Avraham et al. reported improved surgical outcomes and increased dental rehabilitation rates in cases using a virtual surgical plan for mandibular reconstruction [21]. Prefabricated cutting and positioning guides allow creation of a more accurate OCFF contour to reproduce the original mandible. However, repositioning of the mandibular condyle after mandible resection or splitting is also an important factor for a successful clinical outcome. This factor depends on appropriate removal of the bone interference and reproducing the original position of the mandible [11,16,25,26]. In the present study, mandible reconstructions were performed using 3D printed surgical guides composed of cutting, positioning guides, and anchoring units based on virtual surgical simulation. The accuracy of 3D surgical guides was assessed based on stability of the mandibular condyle. Changes in the positions of anatomic landmarks between the planning stage (T0), and immediately after surgery (T1), were defined as reproducibility of the surgical guide for mandibular reconstruction. There were no significant differences between T0 and T1, according to flap type, in reproducing the original position of the mandibular condyle and midline. However, two points (CL and CM) on the condyle showed significantly more change, compared to the midpoint of the mandible (Me), especially in the mediolateral direction for OCFF and in distance (*D*) for FFF. In FFF, Me showed less than 2 mm of error, compared with the planned position, whereas the condyle showed more than 2 mm of error (Table 2 and Figure 4). For intraoperative positioning of the OCFF in mandibular defect, the flap was fixed at a maximum intercuspation of occlusion that could be easily assessed. Conversely, the condylar position could not be thoroughly confirmed, due to limited visual accessibility. Therefore, in our cases, there might be enlarged error on the condyle compared with the midline of the mandible caused by intraoperative intermaxillary fixation for flap positioning. In addition, the slender dimension of FFF might result in more prominent error than the DCIA group.

Overall, OCFF was displaced in the mediolateral direction of the condyle lateral pole for 6 months after surgery, but significant movement of the entire condyle center was within 2 mm. DCIA maintained landmark positions for six months. However, significant movements were observed in most landmarks with FFF. This was especially true in the center of the condyle, up to 2.85 mm, and midpoint of the mandible, 1.56 mm in superoinferior direction (Table 3). In the present study, condyles of the FFF group had moved in the mediolateral direction 6 months after surgery. Several factors explain this finding. Displacement can be affected by both the position and placement of the segment [16]. Masticatory muscles that are resected or peeled off during surgery can interfere with balance of the muscular system. Excessive fibula shaping can lead to bone deficiency, and the condyle may be pulled forward to fill the gap. The remaining condyle is small and deep, which poses a great challenge to fixation [11]. In addition, excessive manipulation can cause edema in the temporomandibular joint [25]. Bone interference between osteotomy gaps may be another factor contributing to condyle displacement in a relaxed state under general anesthesia [3,27].

Displacement of the mandibular condyle may occur as healing progresses between bone segments. Moreover, the force generated by mandibular function induces bone movement that bends and breaks the metal plate. Condylar position can also be severely displaced during healing after reconstruction with FFF [11,28,29]. In contrast, DCIA has an advantage in contours similar to the natural shape of the hemimandible, and is suitable for use in the ipsilateral reconstruction of the mandibular angle or body defects [7,10]. Chen et al. reported that DCIA provides a larger amount of bone that could match with the original mandible dimensions to allow for faster bony union and later osteointegration compared to FFF [3]. Kang et al. suggested that thickness should be considered when using OCFF for mandible reconstruction. DCIA is an alternative option to FFF when there is instability from severe discordance in dimensions between FFF and the original mandible [11]. In our results, DCIA is superior in reproducibility of 3D surgical guide and postoperative stability compared to FFF. The advantageous dimension of DCIA will be helpful to reproduce the original mandible position using a 3D surgical guide, and the condylar position can be stably maintained due its larger dimensions than FFF.

Han et al. reported condyles were laterally displaced about 1.3 mm after sagittal split ramus osteotomy of mandibles, but they effectively regained their original position with semi rigid fixation [26]. They concluded that semi rigid fixation provides micromovement between bone segments to allow functional adjustment of the condylar position. Although SSRO and mandible reconstruction with OCFF differ in the extent of intraoperative condyle manipulation, the result of Han et al.’s study indicates that condylar displacement in DCIA ranged from 1.31 to 1.56 mm, which might be physiologically adjusted during the functional period. However, in FFF with more than 2 mm of condylar displacement, careful intraoperative flap fixation should be taken in addition to closed observation for complications that may occur during mastication, such as plate fatigue fracture, segment breakage, and condyle sagging [11,29].

There are some limitations in this study. The sample size was relatively small and assignment of patients to each group was haphazard under retrospective study design. Therefore, in the present study, it cannot be easily concluded which type of flap would be advantageous in mandible reconstruction using 3D surgical guide, and whether the use of 3D surgical guide would be more accurate compared with cases without 3D guide. Further prospective study is required with a greater sample size and a comparison of other cases without a 3D guide for widespread clinical application of the 3D surgical guide.

## 5. Conclusions

In the present study, total condyle errors were significantly larger than those of the midpoint of the mandible with OCFF. DCIA was superior to FFF in condyle position and midpoint after 6 months of healing, and had advantageous dimensions for the reproducibility of the 3D surgical guides for mandible reconstruction. However, further study should be considered for the clinical application of the 3D surgical guide. Additionally, special consideration should be given in all stages of intraoperative fixation and functional loading when reconstructing mandibles with FFF.

## Figures and Tables

**Table 1:** Patient characteristics.

No.	Age	Gender	Diagnosis	Type of OCFF
1	26	F ^1^	Ameloblastoma	DCIA
2	27	M ^2^	Keratocystic odontogenic tumor	DCIA
3	40	M	Osteosarcoma	FFF
4	41	F	Ameloblastoma	DCIA
5	49	M	Osteomyelitis	DCIA
6	51	M	Ameloblastoma	DCIA
7	52	M	Ondontogenic myxoma	DCIA
8	57	M	Ameloblastoma	DCIA
9	59	M	Keratocystic odontogenic tumor	DCIA
10	65	M	MRONJ ^3^	FFF
11	71	F	Squamous cell carcinoma	FFF
12	75	F	MRONJ	FFF
13	80	F	Osteomyelitis	DCIA
14	83	M	Squamous cell carcinoma	FFF
15	89	F	MRONJ	DCIA

^1^ M: Male; ^2^ F: Female; ^3^ MRONJ: Medication related osteonecrosis of the jaw.

**Table 2:** Reproducibility of 3D surgical guides, change (mm) in positions of landmarks between immediate postoperative (T1) and preoperative stage (T0) in operated side of mandible. There were no significant changes of landmarks between T1 and T0 according to flap type (*p* > 0.05).

Landmark	OCFF	DCIA	FFF
	n = 15	n = 10	n = 5
	Mean (SD)	Mean (SD)	Mean (SD)
**CL**			
*x*	1.44 (0.98)	1.55 (1.09)	1.21 (0.76)
*y*	1.37 (1.54)	1.08 (1.45)	1.95 (1.72)
*z*	0.74 (0.71)	0.73 (0.73)	0.77 (0.76)
*D*	2.42 (1.55)	2.35 (1.50)	2.56 (1.81)
**CM**			
*x*	1.14 (0.95)	1.25 (0.93)	0.92 (1.07)
*y*	0.96 (0.77)	0.83 (0.59)	1.23 (1.07)
*z*	0.73 (1.32)	0.43 (0.54)	1.32 (2.20)
*D*	1.99 (1.40)	1.79 (0.82)	2.40 (2.25)
**CC**			
*x*	1.26 (0.89)	1.38 (0.90)	1.04 (0.93)
*y*	1.06 (1.02)	0.86 (0.75)	1.47 (1.44)
*z*	0.71 (0.84)	0.55 (0.43)	1.04 (1.36)
*D*	2.05 (1.22)	1.91 (0.86)	2.33 (1.83)
**Me**			
*x*	0.48 (0.47)	0.42 (0.41)	0.61 (0.60)
*y*	0.86 (1.20)	0.62 (0.98)	1.33 (1.58)
*z*	0.41 (0.58)	0.19 (0.22)	0.85 (0.83)
*D*	1.17 (1.32)	0.85 (1.02)	1.80 (1.74)

**Table 3:** Stability of condylar position, changes between immediate postoperative stage and six months after surgery in operated side of mandible. Positional changes of landmarks between T2-T0 ^a^ and T1-T0 ^b^ to assess condylar stability.

Landmark	OCFF	DCIA	FFF
	n = 15	n = 10	n = 5
	Mean (SD)	Mean (SD)	Mean (SD)
**CL**			
*x*	1.44 (1.87) *	1.07 (1.43)	2.18 (2.57)
*y*	0.98 (1.25)	0.52 (0.74)	1.90 (1.63)
*z*	0.27 (0.33)	0.24 (0.33)	0.35 (0.34)
*D*	2.03 (2.02)	1.33 (1.54)	3.42 (2.31)
**CM**			
*x*	1.56 (1.70)	1.25 (1.31)	2.20 (2.35) *
*y*	0.60 (0.62)	0.56 (0.44)	0.69 (0.94)
*z*	0.38 (0.48)	0.35 (0.52)	0.42 (0.42)
*D*	1.95 (1.61)	1.56 (1.29)	2.71 (2.06)
**CC**			
*x*	1.45 (1.8)	1.08 (1.39)	2.18 (2.44) *
*y*	0.68 (0.91)	0.48 (0.56)	1.08 (1.39)
*z*	0.3 (0.28)	0.27 (0.28)	0.36 (0.31)
*D*	1.82 (1.85) *	1.31 (1.43)	2.85 (2.32) *
**Me**			
*x*	0.36 (0.45)	0.23 (0.19)	0.61 (0.70)
*y*	0.99 (1.77)	0.47 (0.39)	2.02 (2.95)
*z*	1.12 (1.53)	0.91 (1.07)	1.56 (2.30) *
*D*	1.74 (2.23)	1.13 (1.07)	2.97 (3.47)

^a^ Between 6-month follow-up and preoperative stages; ^b^ Between immediate postoperative and preoperative stages; * *p* < 0.05

## Supplementary Materials

The following are available online at https://www.mdpi.com/1996-1944/13/10/2333/s1, Table S1: Intra-rater and inter-rater reliability, Table S2: Changes (mm) in positions of landmarks between immediate postoperative (T1) and preoperative stage (T0) in intact side of mandible.
