# Supplementary material for: Evaluation of Effective Condyle Positioning Assisted by 3D Surgical Guide in Mandibular Reconstruction Using Osteocutaneous Free Flap
Source: Materials (Basel). 2020 May 19;13(10):2333. doi: 10.3390/ma13102333 (PMC7287575; doi:10.3390/ma13102333)
Supplement: Supplementary file 1 [file materials-13-02333-s001.pdf]

*Supplementary Materials*

# Evaluation of Effective Condyle Positioning Assisted by 3D Surgical Guide in Mandibular Reconstruction Using Osteocutaneous Free Flap

**Seong Ryoung Kim [1], Sam Jang [2], Kang-Min Ahn [1] and Jee-Ho Lee [1,*]**

[1] Department of Oral and Maxillofacial Surgery, College of Medicine, University of Ulsan, ASAN MEDICAL CENTER, Seoul 05505, Korea; alwaysyouth37@gmail.com (S.R.K.); ahnkangmin@hanmail.net (K.-M.A.)
[2] Coreline Soft, Seoul 03991, Korea; semyeong.jang@corelinesoft.com
* Correspondence: jeehoman@amc.seoul.kr or jeehoman@gmail.com; Tel.: +82-2-3010-1757

**Table S1.** Intra-rater and inter-rater reliability.

| Landmark | Intra-Rater Reliability | | | Inter-Rater Reliability | | |
|---|---|---|---|---|---|---|
| *x*-coordinate | | | | | | |
| CL | 0.987 | (0.979 | , 0.999) | 0.996 | (0.982 | , 0.999) |
| CM | 0.998 | (0.995 | , 0.999) | 0.998 | (0.994 | , 0.999) |
| CC | 0.998 | (0.994 | , 0.999) | 0.997 | (0.991 | , 0.999) |
| ME | 0.998 | (0.993 | , 0.999) | 0.986 | (0.959 | , 0.999) |
| *y*-coordinate | | | | | | |
| CL | 0.999 | (0.999 | , 1.000) | 0.989 | (0.989 | , 1.000) |
| CM | 0.989 | (0.989 | , 1.000) | 0.999 | (0.999 | , 1.000) |
| CC | 0.999 | (0.999 | , 0.999) | 0.989 | (0.989 | , 0.999) |
| ME | 0.999 | (0.999 | , 0.999) | 0.999 | (0.999 | , 0.999) |
| *z*-coordinate | | | | | | |
| CL | 0.997 | (0.987 | , 0.999) | 0.997 | (0.990 | , 0.999) |
| CM | 0.987 | (0.981 | , 0.999) | 0.998 | (0.995 | , 0.999) |
| CC | 0.998 | (0.994 | , 0.999) | 0.998 | (0.995 | , 0.999) |
| ME | 0.997 | (0.989 | , 0.999) | 0.998 | (0.993 | , 0.999) |

ICC and 95% confidence interval

**Table S2.** Changes (mm) in positions of landmarks between immediate postoperative (T1) and preoperative stage (T0) in intact side of mandible.

| Landmark | OCFF | DCIA | FFF |
|---|---|---|---|
| | n = 15 | n = 10 | n = 5 |
| | Mean (SD) | Mean (SD) | Mean (SD) |
| **CL** | | | |
| *x* | 0.76 (0.60) | 0.66 (0.46) | 0.96 (0.36) |
| *y* | 0.38 (0.31) | 0.40 (0.36) | 0.33 (0.21) |
| *z* | 0.20 (0.30) | 0.21 (0.36) | 0.36 (0.33) |
| *D* | 1.02 (0.50) | 0.96 (0.39) | 1.12 (0.39) |
| **CM** | | | |
| *x* | 0.65 (0.48) | 0.50 (0.47) | 0.93 (0.54) |
| *y* | 0.39 (0.19) | 0.42 (0.19) | 0.18 (0.18) |
| *z* | 0.44 (0.48) | 0.48 (0.56) | 0.24 (0.28) |
| *D* | 1.03 (0.42) | 0.99 (0.45) | 1.02 (0.53) |
| **CC** | | | |
| *x* | 0.67 (0.49) | 0.54 (0.44) | 0.78 (0.61) |
| *y* | 0.27 (0.23) | 0.32 (0.25) | 0.35 (0.50) |
| *z* | 0.30 (0.30) | 0.33 (0.32) | 0.38 (0.43) |
| *D* | 0.87 (0.48) | 0.79 (0.46) | 1.10 (0.62) |
